# Optical scan and 3D printing guided radiation therapy – an application and provincial experience in cutaneous nasal carcinoma

**DOI:** 10.1186/s41205-022-00136-w

**Published:** 2022-03-29

**Authors:** Jui Chih Cheng, Arbind Dubey, James Beck, David Sasaki, Ahmet Leylek, Shrinivas Rathod

**Affiliations:** 1 Department of Radiation Oncology, CancerCare Manitoba, Winnipeg, Manitoba Canada; 2 Department of Medical Physics, CancerCare Manitoba, Winnipeg, Manitoba Canada

**Keywords:** Optical scan, 3D printing, Skin cancers

## Abstract

**Background:**

Single field Orthovoltage radiation is an acceptable modality used for the treatment of nasal cutaneous cancer. However, this technique has dosimetric pitfalls and unnecessary excessive exposure of radiation to organs at risk (OAR). We present the clinical outcome of a case series of cutaneous nasal tumours using a novel technique incorporating an optical scanner and a 3-dimensional (3D) printer to deliver treatments using parallel opposed (POP) fields.

**Materials and methods:**

The POP delivery method was validated using ion chamber and phantom measurements before implementation. A retrospective chart review of 26 patients treated with this technique between 2015 and 2019 was conducted. Patients’ demographics and treatment outcomes were gathered and tabulated. These patients first underwent an optical scan of their faces to collect topographical data. The data were then transcribed into 3D printing algorithms, and positive impressions of the faces were printed. Custom nose block bolus was made with wax encased in an acrylic shell; 4 cm thick using the printed face models. Custom lead shielding was also generated. Treatments were delivered using 250 KeV photons POP arrangement with 4 cm diameter circle applicator cone and prescribed to the midplane. Dose and fractionation were as per physician discretion.

**Results:**

Phantom measurements at mid-plane were found to match the prescribed dose within ±0.5%. For the 26 cases in this review, the median age was 78.5 years, with 15 females and 11 males. 85% of cases had Basal cell carcinoma (BCC); 1 had squamous cell carcinoma (SCC), one had synchronous BCC + SCC, and 1 had Merkel cell carcinoma. Twenty-one cases had T1N0 disease, 4 had T2N0, and 1 had T3N0. Dose and fractionation delivered were 40Gy in 10 fractions for the majority of cases. The complete response rate at a median follow-up of 6 months was 88%; 1 patient had a refractory tumour, and one patient had a recurrence. Toxicities were minor with 81% with no reported side effects. Three patients experienced grade 3 skin toxicity.

**Conclusions:**

Utilization of optic scanner and 3D printing technology, with the innovative approach of using POP orthovoltage beams, allows an effective and efficient way of treatment carcinomas of the nose with a high control rate and low toxicity profiles.

## Introduction

Cutaneous malignancies are the most common cancer, with basal cell carcinoma (BCC) and squamous cell carcinoma (SCC) making up 95% of the cases [1]. Therefore, surgical resection with adequate margins is often employed as the first treatment modality [2]. However, radiotherapy (RT) is also invaluable, especially in poor surgical candidates and challenging anatomical sites.

The use of superficial X-rays to treat superficial nonmelanoma skin cancer is widely established [3–8]. However, treatment of such cancers of the face, particularly on the nose, can be challenging. Traditionally, nonmelanoma skin cancers in this location are treated with a single anterior field using ortho-Voltage or electron beam radiation [9]. However, delivering a homogeneous dose to the target can often be difficult due to the contour variability. Furthermore, the underlying normal tissues like the oral cavity, bones, nasopharynx and other organs at risk (OAR) are exposed to radiation unnecessarily, resulting in associated side effects.

An approach that could potentially minimize unnecessary exposure of underlying structures to radiation would be to treat the lesion laterally with two parallel opposed (POP) fields. Despite using opposing lateral fields, dose homogeneity remains challenging due to the contour of the nasal region [9]. To mitigate this, we typically used a customized rectangular nose bolus. This was traditionally done at our institution using plaster of Paris mold to first generate a negative impression mold with the patient lying supine. Subsequently, a positive face model was then created. Wax block bolus was finally created using this positive impression of the patient’s face. While effective, this method can be extremely difficult or impossible with patients that are claustrophobic or medically unable to lie supine. It is also resource-intensive and requires long patient appointment times.

3-dimensional (3D) printing offers an effective alternative to traditional molding and has been implemented clinically, including surgery, customized implants and radiotherapy boluses [10–14]. To print customized accessories, accurate topographical data of patient anatomies is required. Computed tomography (CT) is an excellent modality in acquiring accurate topographical data of patient anatomies [13, 15]. However, CT scans can be resource-intensive and subject patients to radiation exposure. Commercially available optical scanners at relatively low cost provide an excellent alternative to CT scans. It can generate both topographical and textural information of the patients [16]. These data can then be used to create 3D face models and subsequently customized treatment accessories. At our institution, it is standard of care to employ both the optical scanner and 3D printer technology to generate the accessories required for radiation delivery. We previously showed successful applications of both optical scanners and 3D printers for creating customized bolus and lead shields in contour-challenging areas [16, 17].

The nose is a common site for non-melanomatous skin cancer. However, few studies are investigating the application of 3D printing in radiation treatments of nasal cutaneous cancers [12, 13, 18, 19]. Furthermore, of the studies available, none used the optical scanners or provided insights on the clinical outcomes. In the present study, we highlight our applications of the optic scanner and 3D printers to create the custom nose block bolus and the clinical outcomes from this novel treatment.

## Material and methods

The local research ethics board approved this retrospective study. Between November 30th, 2015 and May 22nd, 2019, 26 patients with nonmelanoma cutaneous carcinoma of the nose who received radiotherapy using customized 3D nose block were identified using the electronic medical record (EMR) database (ARIA, Varian Medical Systems).

### Patient evaluation, treatment delivery, and follow up

Patients were initially evaluated in the clinic by a radiation oncologist, and clinical targets were outlined manually with a marker as per the clinician’s discretion. Optical scans and 3D print of the patient’s face were used to generate the customize nose block bolus (Described above).

With the completion of the nose blocks, patients were invited to the treatment room. In a supine position, a lead shield and nose block were placed on the patient. Cone positions were marked directly on the nose block. 250kVp photons treatments were delivered using POP (Fig. 3) sing OrthoVoltage™ with 100% dose prescribed to 2 cm depth. Doses between 36Gy in 6 fractions to 55Gy in 22 fractions were delivered at physicians’ discretion. Patients were followed post-treatment at least once to assess treatment response. Any side effects were recorded in the charts and were graded per CTCAEv5.0 criteria on Radiation Dermatitis.

### Data collection and analysis

Demographic parameters (age at diagnosis, gender and tumour / treatment-related parameters (staging, histology treatment intent, dose, fractionation, energy, first/last dates of treatments, and outcomes (clinical outcome and toxicity) were extracted from EMR and tabulated. Results are expressed in percentage (%) of the total study cohort. Validation of beam data is represented in graph format.

## Results

### Optical scanner

The optical scanner used in this study has been described in other studies by our centre [16, 17]. Briefly, an optical scanner (Sense 3D Scanner, 3D Systems, USA) utilizes both optical and infrared cameras, along with an infrared source to gather both topographical and textural data. The data handling by proprietary hardware within the scanner is described elsewhere [20, 21]. The details on commissioning of this device from our centre are available elsewhere [16]. A gantry was made from a circular aluminum tube diameter 126.5 cm to mount the optical scanner. The scanner can be moved isocentrically along the loop with an adjustable scanner to surface distance and allows a +/− 20degrees tilt with respect to the patient table. During the scan, the scanner maintains a constant distance while scanning, allowing gathering all data in one pass and customizing scanner orientation to patient anatomy. The scans are then imported into a mesh editing software (MeshMixer, Autodesk) to amend any artifacts and scale to the desired volume for printing. The file is then exported to an open-source program (Slic3r) to generate the ‘g-code’ – a programming language that controls the 3D printer.

### 3D printing and nose block generation

A positive impression of the face scanned was printed using the MakerGear M2 3D printer (MakerGear, USA) with ColorFabb PLA/PHA filaments (ColorFabb, The Netherlands). The commissioning of the printer for treatment purposes is described in previous publications [16, 17]. The printer and filament characteristics are described by Sasaki et al. (2019) [17]. The printer lays down 0.3 mm layers to topographically produce the face models. The infill settings are optimized to produce a durable product. The quality of printings was optimized by choosing a slow print speed (typically at 30 mm/second. The typical print time is 12 h per model.

The resulting model is used to generate treatment accessories: a lead shield, described in detail by Sharma et al. (2018) [16], and a nose block bolus. The nose block is made as follows: a rectangular acrylic shell 4 cm thick (other dimensions customized to patient anatomy) is first made, and hot wax is poured into the shell to make a rectangular bolus. Using the face model as a guide, the bottom of the wax block is then carved manually to create a negative impression of the patient’s nose. 4 cm circular field is marked directly on the lateral surfaces of the nose block to easily replicate treatment setups.

### Percentage depth dose (PDD), beam profile and point dose verifications

The dose deposition by a 250kVp beam collimated with a 4 cm diameter 30 cm FSD circular applicator may vary between Orthovoltage units. The percent depth dose (PDD) and beam profile were measured to characterize treatment uniformity in a homogeneous water phantom. Measurements were acquired at depth using a PTW MP3 scanning tank equipped with PTW semi-flex ionization chambers (model 31,010). Surface measurements were performed using a PTW Advanced Markus chamber (model 34,045) in solid water. To confirm consistency, PDD measurements were compared to the historical BJR 25 standard, with corrections for FSD differences applied according to the methodology suggested by Burns (from BJR 25) [22]. Finally, an end-to-end wax phantom was constructed to fit a calibrated Exradin ionization chamber (model A12) and used to measure the dose rate at the prescription point at 2 cm depth after exposure to a POP beam arrangement.

### Dosimetric validation

**Fig. 1:**
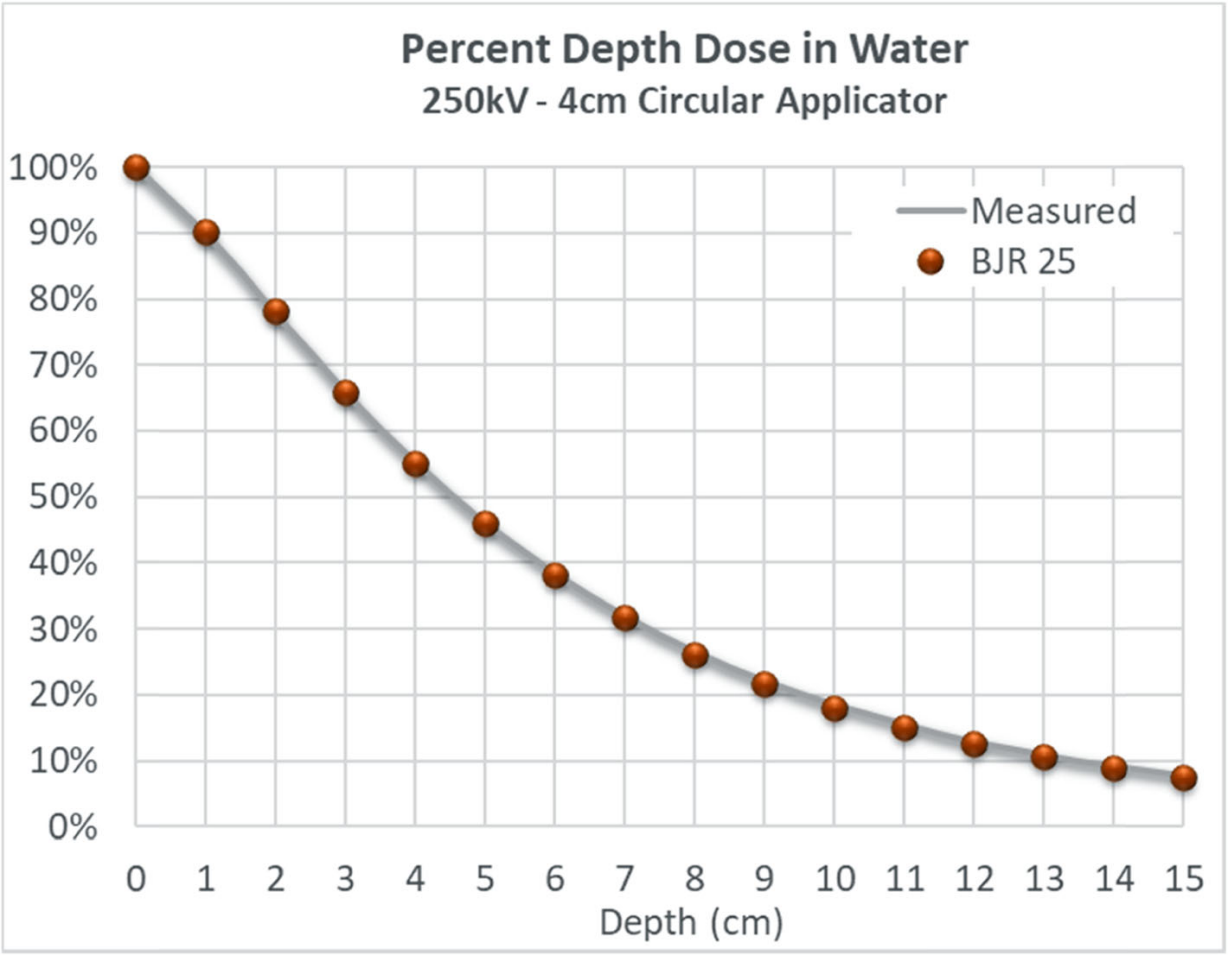
Institute verification of percentage depth dose of 250KeV photon beam in water phantom

**Fig. 2:**
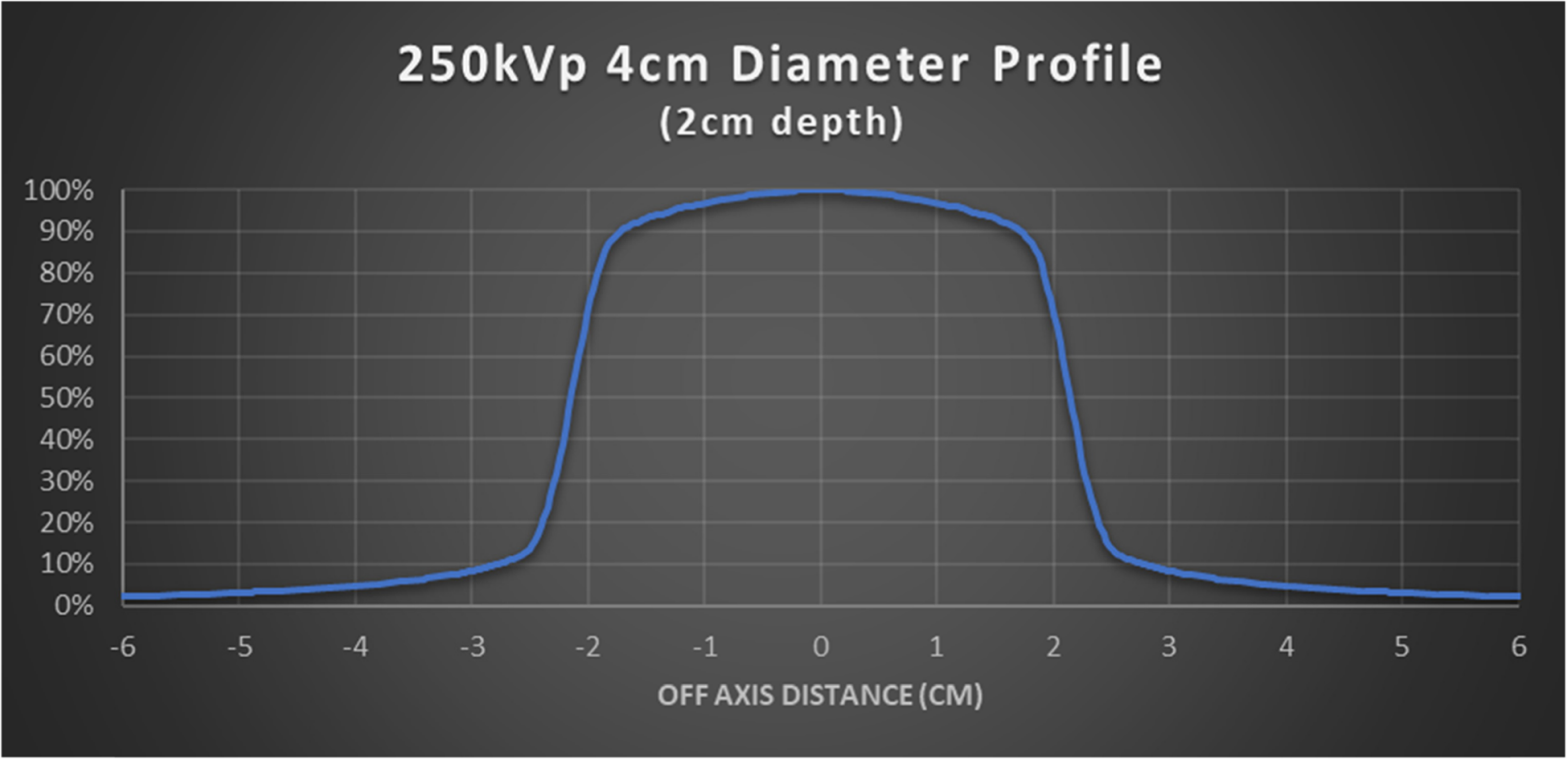
Beam profile of 250KeV photon at 2 cm depth of wax

**Fig. 3:**
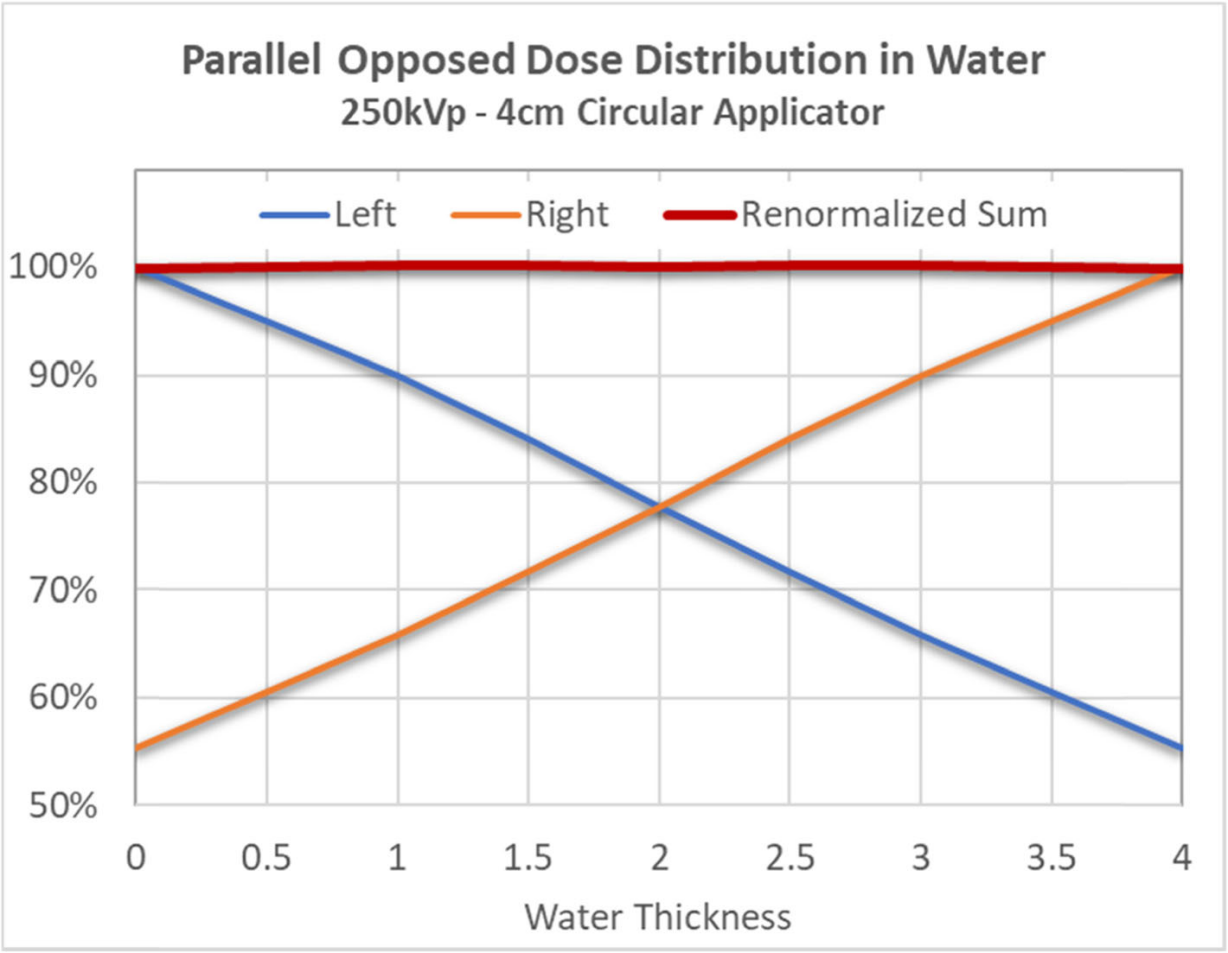
Parallel opposed Dose Distribution of 250KeV photons in water phantom

Figure 1 depicts the 250kVp PDD in water, as collimated by a 4 cm diameter 30 cm FSD applicator. Measured points agree with the corrected BJR standard within ±0.5%. Fig. 3 illustrates the approximate dosimetric uniformity that could be achieved across a 4 cm thick homogeneous water phantom using the right and left POP PDD combination outlined in this report. Dosimetric uniformity across field width is illustrated by the beam profile shown in Fig. 2.

The dose output of the orthovoltage unit was determined using “in-air” calibration according to the TG61 protocol [23]. Based on the PDD measurements outlined above and assuming water equivalency of our wax phantom, the predicted dose rate at the prescription point was calculated to be 1.84 cGy/MU. Chamber measurements gave an experimental value equal to 1.83 cGy/MU, exhibiting agreement within ±0.5% and validating our methodology.

### Clinical data

Patient demographics, diagnosis, and treatment data were summarized in Table 1. The Median follow-up time was six months. The median age at diagnosis was 78.5 years (range 58 to 91 years). Of the 26 patients, 15 were females (58%), and 11 were males (42%). Basal cell carcinoma was the most common histology accounting for 22 (85%) patients, followed by squamous cell carcinoma 2 (7.7%), Merkel cell carcinoma 1 (3.8%), concurrent Basal cell and squamous cell carcinomas 1 (3.8%) in the same treatment field.

**Table 1:** Patient demographics

***N*** = 26		%
Sex		
Male	11	58
Female	15	42
Histology		
BCC	22	84
SCC	2	8
Merkel Cell	1	4
BCC + SCC	1	4
Stage		
T1N0	21	80
T2N0	4	16
T3N0	1	1
Treatment intent		
Curative	21	80
Adjuvant	3	12
Salvage	1	4
Palliative	1	4
Dose/Fraction		
35Gy/5#	4	15
36Gy/6#	3	12
40Gy/10#	17	65
50Gy/20#	1	4
55Gy/22#	1	4

The vast majority, 21 (81%), of patients had T1N0 disease, followed by four patients with T2N0 and one patient with T3N0 carcinoma. Beam energy of 250kVp was used for all treatments. Seventeen patients (65%) were treated with curative intent using 4000 cGy in 10 fractions. The single patient (3.8%) with Merkel cell carcinoma was treated with 5500 cGy/22fraction.

Two (7.7%) patients were treated in an adjuvant setting for post-operative SCC and BCC using 4000 cGy in 10 fractions. Of all treated patients, 23 (88%) had complete treatment responses. 1(BCC, T1N0) had recurred disease, 1(SCC, T2N0) had refractory treatment (Table 2).

**Table 2:** Treatment outcome

	N	%
Complete response	23	88 (92)**
Recurred*	1	4
Refractory*	1	4
Lost in follow up	1	4

**92% complete response rate if lost in follow up was excluded from analysis

Excluding the patient who was lost in follow-up, the complete treatment response rate is 92%. In the recurrent/refractory cases, lesions were treated with Moh’s surgery plus flap reconstructions. One patient (Merkel cell carcinoma) was lost in follow-up. 21 (81%) patients did not report any toxicity. Grade 1 and grade 3 cutaneous side effects were reported in 2 and 3 patients, respectively. (Table 3).

**Table 3:** Toxicity Profile

	N	%
Non reported “No issues”	21	81
Grade 1	2	8
Grade 2	0	0
Grade 3	3	12
Grade 4	0	0

## Discussion

The nose is a common site for non-melanomatous skin cancer. However, there are few studies investigating the application of 3D printing in radiation treatments of nasal cutaneous cancers [12, 13, 18, 19]. In the present study, we highlight our applications of the optic scanner and 3D printers to create the custom nose block bolus and the clinical outcomes from this novel treatment.

We found the use of optical scans and 3D printing has several benefits in nasal carcinoma treatment. The use of an optical scanner to acquire treatment planning data avoids the need for CT simulation scans. The optical scanner uses infrared and visible lights to acquire data sparing the patient from ionizing radiation exposures. 3D printed face allows custom nose blocks to be created without requiring patients to be in the hospital during this process. Optical scans and 3D printing produce facial contours with high accuracy; thus, nose blocks can be customized to a high degree of conformity to patients’ unique anatomies. Given the nose block is customized to individual patients with pre-marked fields, treatment setups are quick and highly reproducible. The clinical setup can be reproduced consistently, and treatment simulation or image verifications are not required. This saves staffing resources and reduces inter-fraction uncertainties.

The nose is a challenging treatment site to treat due to the highly variable contours and could have 2 cm or more in variations from the tip of the nose to nasal folds. A single anterior field would typically create dose heterogeneity across the nose. Furthermore, structures underneath the target, including oral mucosa, gingiva and teeth, are exposed to higher doses of radiation. Dosimetric challenges from the nose contour have been recognized in the literature. Currie et al. (2007) conducted a dosimetric study on a nose phantom using both single anterior field and laterally opposed approach [9]. In their study, there is a significant dose drop off from depth of 6.5 mm to 21.5 mm, from 84% to 50%, respectively [9]. This depth dose profile is not ideal if the whole nose needs to be included in the clinical volume; to ensure adequate dose at depth, hot spots will be created at the tip of the nose, which could contribute significantly to the skin toxicity. The authors also employed a lateral approach by placing a cone laterally and perpendicular to the nose phantom; This approach resulted in 27% underdosing of the nose at the midplane [9]. The authors postulated that this is due to the fact that the cone could not be applied directly to the side of the nose and required an offset of 1 cm, which leads to dose reduction; the small backscatter volume of the nose itself was also a contributor in this study [9]. This problem is effectively solved by applying the nose block, shaping the nose essentially into a homogeneous, tissue-equivalent box. The treatment cone can be applied directly to the nose block, and the POP field arrangement provides a homogenous dose across the target. This concept is validated in our phantom measurements, shown in Figs. 1, 2 and 3. The POP approach also effectively spares the underneath normal tissues; the advantage is recognized by Currie et al. (2007) [9]. To our knowledge, there is currently no published literature that utilizes this technique.

In the review of indications, outcomes of radiation treatment of skin cancers, Wang et al. (2009) reported high local control rates in BCC treatment with low complications rates [1]. Duinkerken et al. (2016) demonstrated in their single-centre retrospective study showed excellent treatment response in treating BCC with overall local control rates of 96.3% at five years post treatment [8]. Though the study only has a small cohort of 26 people. We have observed a 92% complete response rate by six months post-treatment. This is consistent with the control rates we have seen in our literature reviews. We have also observed a low rate of acute toxicities: only three patients had grade 3 skin toxicities, and two patients had grade 1 toxicities. Duinkerken et al. (2016) reported a 75% grade 2 or higher toxicities, which is much higher than our observation [8]. Most of the patients reported “no issues” on follow-up (Table 3); it is possible that mild dermatitis is underreported in our cohort. Late toxicities were not characterized due to the length of follow-up in this study (median of 6 months). This suggests that outcome and toxicity data are consistent with reported literature and do not result in a higher risk of local failures.

Despite uniqueness, there are several limitations to our current study. The use of nose block and the POP field arrangement limit the treatment sites to the apex and nasal alae, as these structures are the ones centred in our 4 cm cone fields. This setup can not be used to treat lesions further up the dorsum nor the bridge. The nose fitting side of the wax blocks was carved manually; this leaves the nose block vulnerable to small air gaps and misalignments between the nose block and nasal surfaces, and the quality and reproducibility of the nose blocks are highly dependent on the staff skills. Such an issue was also highlighted in other studies [14, 19]. This particular problem can be mitigated by 3D printing the entire nose block using the topographical data of the nose collected by the optical scanner. In their dosimetric study of 3D printed nose bolus, Albantow et al. (2020) demonstrated superior conformity to the nose when the bolus was 3D printed as opposed to manually created [19]. In this case series, our cohort is only made up of 26 patients. Furthermore, it is a retrospective study without control groups. The utility of our technique could only be presented semi-quantitatively.

## Conclusion

Utilization of optic scanner and 3D printing technology along with innovative approach of using POP orthovoltage beams allows effective and efficient way of treatment carcinomas of the nose with high control rate and low toxicity profiles.

## Data Availability

Deidentified data available on request, as per local policy.

## References

[1] Wang Y, Wells W, Waldron J (2009). Indications and outcomes of radiation therapy for skin cancer of the head and neck. Clin Plast Surg.

[2] Avril M-F, Auperin A, Margulis A, Gerbaulet A, Duvillard P, Benhamou E, Guillaume JC, Chalon R, Petit JY, Sancho-Garnier H, Prade M, Bouzy J, Chassagne D (1997). Basal cell carcinoma of the face: surgery or radiotherapy? Results of a randomized study. Br J Cancer.

[3] Rong Y, Zuo L, Shang L, Bazan JG (2015). Radiotherapy treatment for nonmelanoma skin cancer. Expert Rev Anticancer Ther.

[4] Morrison WH, Garden AS, Ang KK (1997). Radiation therapy for nonmelanoma skin carcinomas. Clin Plast Surg.

[5] Veness M, Richards S (2003). Role of modern radiotherapy in treating skin cancer. Australas J Dermatol.

[6] Childers BJMD, Goldwyn RMMD, Ramos DMD, Chaffey JMD, Harris JRMD (1994). Long-term results of irradiation for basal cell carcinoma of the skin of the nose. Plast Reconstr Surg.

[7] Thom GA, Heywood JM, Cassidy B, Freund JM (2003). Three-year retrospective review of superficial radiotherapy for skin conditions in a Perth radiotherapy unit. Australas J Dermatol.

[8] Duinkerken CW, Lohuis PJFM, Heemsbergen WD, Zupan-Kajcovski B, Navran A, Hamming-Vrieze O, Klop WMC, Balm FJM, al-Mamgani A (2016). Orthovoltage for basal cell carcinoma of the head and neck: excellent local control and low toxicity profile. Laryngoscope.

[9] Currie M, Bailey M, Butson M (2007). Verification of nose irradiation using orthovoltage x-ray beams. Australas Phys Eng Sci Med.

[10] Choi JW, Kim N (2015). Clinical application of three-dimensional printing Technology in Craniofacial Plastic Surgery. Arch Plast Surg.

[11] Succo G, Berrone M, Battiston B, Tos P, Goia F, Appendino P, Crosetti E (2015). Step-by-step surgical technique for mandibular reconstruction with fibular free flap: application of digital technology in virtual surgical planning. Eur Arch Otorhinolaryngol.

[12] Kim S-W, Shin H-J, Kay CS, Son SH (2014). A customized bolus produced using a 3-dimensional printer for radiotherapy. PLoS One.

[13] Burleson S, Baker J, Hsia AT, Xu Z (2015). Use of 3D printers to create a patient-specific 3D bolus for external beam therapy. J Appl Clin Med Phys..

[14] Łukowiak M, Jezierska K, Boehlke M, Więcko M, Łukowiak A, Podraza W, Lewocki M, Masojć B, Falco M (2016). Utilization of a 3D printer to fabricate boluses used for electron therapy of skin lesions of the eye canthi. J Appl Clin Med Phys.

[15] Evans PM (2008). Anatomical imaging for radiotherapy. Phys Med Biol.

[16] Sharma A, Sasaki D, Rickey DW, Leylek A, Harris C, Johnson K, Alpuche Aviles JE, McCurdy B, Egtberts A, Koul R, Dubey A (2018). Low-cost optical scanner and 3-dimensional printing technology to create lead shielding for radiation therapy of facial skin cancer: first clinical case series. Adv Radiat Oncol.

[17] Sasaki DK, McGeachy P, Aviles JEA, McCurdy B, Koul R, Dubey A (2019). A modern mold room: meshing 3D surface scanning, digital design, and 3D printing with bolus fabrication. J Appl Clin Med Phys.

[18] Zhao Y, Moran K, Yewondwossen M, Allan J, Clarke S, Rajaraman M, Wilke D, Joseph P, Robar JL (2017). Clinical applications of 3-dimensional printing in radiation therapy. Med Dosim Off J Am Assoc Med Dosim.

[19] Albantow C, Hargrave C, Brown A, Halsall C (2020). Comparison of 3D printed nose bolus to traditional wax bolus for cost-effectiveness, volumetric accuracy and dosimetric effect. J Med Radiat Sci.

[20] Diaz MG, Tombari F, Rodriguez-Gonzalvez P, Gonzalez-Aguilera D (2015). Analysis and evaluation between the first and the second generation of RGB-D sensors. IEEE Sensors J.

[21] DiFilippo NM, Jouaneh MK (2015). Characterization of different Microsoft Kinect sensor models. IEEE Sensors J.

[22] Central axis depth dose data for use in radiotherapy. A survey of depth doses and related data measured in water or equivalent media. Br J Radiol Suppl. 1983;17:1-147. 6600113

[23] Ma C-M, Coffey CW, DeWerd LA, Liu C, Nath R, Seltzer SM, Seuntjens JP, American Association of Physicists in Medicine (2001). AAPM protocol for 40–300 kV x-ray beam dosimetry in radiotherapy and radiobiology. Med Phys.

